# Modification of Peak Plasticity Induced by Brief Dark Exposure

**DOI:** 10.1155/2019/3198285

**Published:** 2019-09-03

**Authors:** Alexander J. Lingley, Donald E. Mitchell, Nathan A. Crowder, Kevin R. Duffy

**Affiliations:** Department of Psychology & Neuroscience, Dalhousie University, Halifax, NS, Canada B3H 4R2

## Abstract

The capacity for neural plasticity in the mammalian central visual system adheres to a temporal profile in which plasticity peaks early in postnatal development and then declines to reach enduring negligible levels. Early studies to delineate the critical period in cats employed a fixed duration of monocular deprivation to measure the extent of ocular dominance changes induced at different ages. The largest deprivation effects were observed at about 4 weeks postnatal, with a steady decline in plasticity thereafter so that by about 16 weeks only small changes were measured. The capacity for plasticity is regulated by a changing landscape of molecules in the visual system across the lifespan. Studies in rodents and cats have demonstrated that the critical period can be altered by environmental or pharmacological manipulations that enhance plasticity at ages when it would normally be low. Immersion in complete darkness for long durations (dark rearing) has long been known to alter plasticity capacity by modifying plasticity-related molecules and slowing progress of the critical period. In this study, we investigated the possibility that brief darkness (dark exposure) imposed just prior to the critical period peak can enhance the level of plasticity beyond that observed naturally. We examined the level of plasticity by measuring two sensitive markers of monocular deprivation, namely, soma size of neurons and neurofilament labeling within the dorsal lateral geniculate nucleus. Significantly larger modification of soma size, but not neurofilament labeling, was observed at the critical period peak when dark exposure preceded monocular deprivation. This indicated that the natural plasticity ceiling is modifiable and also that brief darkness does not simply slow progress of the critical period. As an antecedent to traditional amblyopia treatment, darkness may increase treatment efficacy even at ages when plasticity is at its highest.

## 1. Introduction

Disruption of normal binocular vision during critical periods early in postnatal development can provoke anatomical and physiological alterations to neurons within the primary visual pathway. Monocular deprivation (MD) by eyelid closure can elicit a shift in cortical responsivity so that most neurons come to be excited only by stimulation of the nondeprived eye [1], leaving the deprived eye able to control few neurons and with a visual acuity deficit, called amblyopia [2], that is most severe in the central visual field [3]. This deprivation-induced shift in ocular dominance is consequent to a reduction in the number and strength of cortical neural connections serving the deprived eye [4–6], which is reflected by a reduction in the cross-sectional soma area of neurons within deprived-eye recipient layers of the dorsal lateral geniculate nucleus (dLGN) in the thalamus [7, 8].

**Figure 1:**
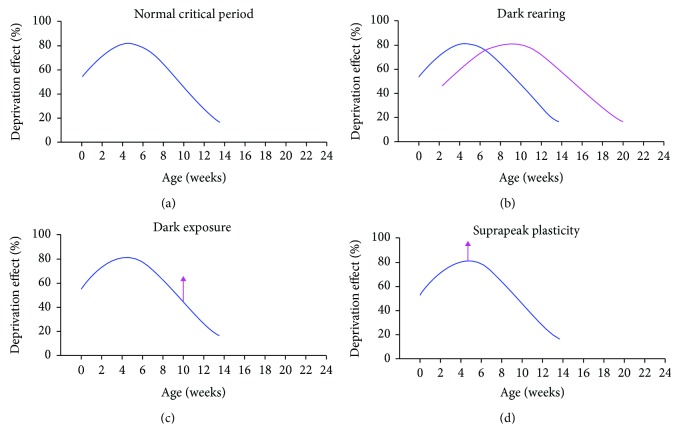
Illustration of the effect on plasticity capacity following immersion in complete darkness. Profile of the critical period in normally reared cats (based on data from [9]) demonstrates peak plasticity at about 4 weeks of age followed by a progressive decline to low levels that are maintained into adolescence (a). Dark rearing from near birth and for long durations has been postulated to slow the overall time course of the critical period (magenta profile in (b)) so that enhanced plasticity can be observed at ages when normally reared animals exhibit lower plasticity capacity (based on [15]). More recently, short durations of darkness (dark exposure) in rodents and cats have been employed to raise plasticity levels beyond that observed from age-matched controls (c). In the current study, we investigated the possibility that 10 days of dark exposure imposed just prior to the critical period peak can enhance plasticity capacity beyond its natural maximum (d).

The capacity of the visual system to be modified by imbalanced visual experience is regulated by age, reaching peak plasticity levels early in the postnatal life and thereafter declining through adolescence and into early adulthood [9–12]. In cats, the critical period for susceptibility to MD (Figure 1(a)) reaches its peak at about 4 weeks of age [9–11] and is then followed by a decline to low levels by about 12-16 postnatal weeks [9, 10] followed again by an even slower decay to negligible levels at about 10 months [11, 12]. The capacity for recovery from the effects of MD likewise adheres to a critical period, but with a shorter timespan and with little recovery observed when MD is followed by reverse occlusion beyond about 12 weeks postnatal [13, 14].

The notion that plasticity capacity is rigidly associated with age is at odds with a growing number of studies on mice, rats, and cats demonstrating that the critical period profile is itself plastic, a concept referred to as *metaplasticity* [16]. Genetic, molecular, and experiential interventions have been employed to alter key critical period parameters that have enabled manipulation of plasticity levels in the visual system [17, 18]. In rodents, critical period timing can be modified through manipulation of GABAergic [19–21] or glutamatergic signalling [22, 23], by alteration of the neurotrophin expression [24] or the expression of protein constituents of the extracellular matrix [25–28], as well as by the tweaking of epigenetic targets [29–31]. In aggregate, these studies demonstrate that plasticity capacity in the visual system can be adjusted beyond what would be available in age-matched normally reared animals.

Immersing young cats in complete darkness has long been known to extend the critical period [32–36] and has provided a means of modifying a variety of neural plasticity regulators to bring about high levels of visual plasticity [37–43]. Kittens reared from near birth in complete darkness maintain sensitivity to MD in the visual cortex even when dark rearing extends to 10 months of age at which time the cortex of normal animals is immutable [33]. A prominent theory of how long durations of darkness (called dark rearing) modify plasticity levels in the visual system postulates that dark rearing slows the time course of the critical period (Figure 1(b)), with both its onset and decay being delayed relative to animals reared under normal conditions [15]. More recent research in mice, rats, and cats has demonstrated that long durations of dark rearing are not necessary to provoke enhanced plasticity capacity and that much shorter durations of darkness (called dark exposure) can significantly elevate plasticity levels in the visual system [39, 41, 44]. The notion that darkness acts to slow the progress of the critical period profile is incongruent with rodent research showing plasticity enhancement following dark exposure in juveniles and adults [39, 44] and also with cat research showing a modest plasticity boost when dark exposure is imposed past the critical period peak (Figure 1(c); [45]). The ability for dark exposure to raise the level of plasticity capacity rather than simply slow its progression implies that there are differences between the mechanisms mediating the effects of long- and short-term dark immersion and suggests that dark exposure does not alter plasticity levels simply by slowing progress of the critical period. In this study, we examined whether the plasticity enhancement conferred by dark exposure occurs when darkness is imposed at the peak of the critical period, a time when plasticity capacity is at its natural maximum (Figure 1(d)). A modification of peak plasticity would indicate that dark exposure does not cause a protraction of the critical period but rather alters the constellation of plasticity-related molecules enabling enhanced plasticity even from its natural maximum. We demonstrate that 10 days of dark exposure applied immediately prior to the peak of the critical period can enhance the effect of a week-long period of MD. These results indicate that dark exposure does not simply slow the temporal progression of the critical period, but is efficacious even when applied within the first postnatal month and can elevate plasticity levels beyond natural limits.

## 2. Materials and Methods

### 2.1. Animals and Rearing Histories

Eight animals were reared from birth in a closed cat breeding colony at Dalhousie University for the purposes of this study. In summary, four animals were monocularly deprived for 7 days at postnatal day 30 (MD-only group), and four animals were immersed in darkness for 10 days from postnatal day 20 to 30 and then removed from darkness and immediately monocularly deprived for 7 days (dark exposure+MD group). All experimental procedures adhered to protocols that were approved by the Dalhousie University Committee on Laboratory Animals in accordance with policies established by the Canadian Council on Animal Care.

### 2.2. Monocular Deprivation

Monocular deprivation was performed under general gaseous anesthetic using 3-4% isoflurane in oxygen. The upper and lower palpebral conjunctivae of the left eye were sutured with vicryl suture material, followed by closure of the overlying eyelids with silk suture, as has been described in detail previously [46]. The surgery lasted approximately 15 minutes after which anesthetized animals were administered a subcutaneous injection of Anafen for postoperative analgesia, as well as topical ophthalmic Alcaine (proparacaine hydrochloride) to mitigate postprocedural discomfort. A broad-spectrum topical antibiotic (1% Chloromycetin) was also given postprocedurally to mitigate infection.

### 2.3. Dark Exposure

Kittens indicated for darkness exposure were housed for 10 days in a darkness facility that has been in use for many decades and has been described in detail previously [47]. In brief, the darkness facility contains three darkrooms accessible only via a series of completely dark anterooms, each segregated by doors sealed at all margins to prevent any entrance of light. The central darkroom is used to house the communal cage containing kittens and their mother, with the dark anterooms used as transfer space to facilitate cleaning and husbandry. Daily feeding, cleaning, and social interaction were provided by experienced technicians. The appearance, health, weight, and well-being of animals in the dark were monitored through the use of a CCD camera and infrared illumination system (>820 nm) that remained off when not in use. Animals destined to be monocularly deprived following 10 days of dark exposure were transported to a nearby surgical suite within an opaque, light-impermeable chamber that was designed to allow for the administration of gaseous anesthetic while mitigating exposure to light.

### 2.4. Histology

Histological procedures were the same for all animals in this study. Kittens were anesthetized with isoflurane (5% in oxygen) and euthanized with an intraperitoneal lethal dose of sodium pentobarbital (Euthanyl; 150 mg/kg). Subsequently, animals were transcardially perfused with 150 mL of phosphate-buffered saline (PBS) followed by 150 mL of 4% dissolved paraformaldehyde in PBS. Brain tissue was immediately extracted following perfusion, and the thalamus containing the dLGN was carefully dissected from the overlying cortex using a scalpel. The block of tissue containing the dLGN was immersed in a PBS solution containing 30% sucrose for cryoprotection. Five days later, sections of the dLGN were sliced into 50 *μ*m thick coronal sections using a freezing microtome (Leica SM2000R; Germany). A portion of the cut sections were stained for Nissl substance by mounting them onto glass sides, immersing them in a graded series of ethanol concentrations, followed by immersion in a solution of 0.1% cresyl violet acetate dye dissolved in distilled water. A separate set of sections was labeled for neurofilament protein via immersion in PBS containing a mouse monoclonal antibody targeting the heavy chain subunit of neurofilament (1 : 1000 dilution; SMI-32; BioLegend, San Diego, CA). Sections were left overnight, then thoroughly washed in PBS, and immersed in a PBS solution containing goat-anti-mouse secondary antibody for 1 hour (1 : 500; Jackson Immunoresearch, West Grove, PA). Following another wash with PBS, sections were placed in an avidin and peroxidase-conjugated biotin solution for one hour (PK6100; Vector Labs, Burlingame, CA). Neurofilament labelling was visualized through reaction with 3,3′-diaminobenzidine. Tissue sections stained for Nissl substance or labelled for neurofilament protein were immersed in a graded series of ethanol concentrations, cleared using Histoclear (DiaMed Lab Supplies Inc.; Canada) and coverslipped using Permount (Fisher Scientific; Canada). Tissue from one MD-only animal and one animal immersed in darkness before MD was fixed suboptimally following perfusion and exhibited pale reactivity for neurofilament within and beyond the dLGN. Although excellent Nissl staining enabled soma size quantification from these animals, the quality of neurofilament labeling was insufficient for quantification so they were excluded from our quantification.

The specificity of the primary antibody, SMI-32 (Table 1), for the nonphosphorylated heavy-chain subunit of neurofilament was verified with an immunoblot of homogenized normal cat primary visual cortex. The labelled blots revealed bands corresponding with the expected mass of NF-H [48].

### 2.5. Quantification

Quantification of neuron soma size and neurofilament immunoreactivity in the dLGN was performed blind to animal rearing condition. Quantification was performed using a BX-51 microscope (Olympus; Markham, Ottawa, Canada) fitted with a DP-70 digital camera (Olympus; Markham, Ottawa, Canada) and a computerized stereology software package (newCast; Visiopharm, Denmark). The cross-sectional area of neuron somata within A and A1 layers of the left and right dLGN was measured from Nissl-stained sections using the “nucleator” stereology probe, whereas the density of neurofilament immunoreactive neurons in separate sections was measured using the “optical dissector” stereology probe. Neurons in Nissl-stained sections were distinguished from glial cells by established selection criteria [1, 7, 8]. Cells characterized by dark cytoplasmic and nucleolar staining with light nuclear staining were considered admissible for quantification. These criteria help to reduce the chance of inadvertently quantifying cell caps, rather than cells cut through the somal midline. Cells within the dLGN labelled for neurofilament and selected for quantification exhibited dark cytoplasmic reactivity with pale or absent labeling within the nucleus. A summary of measurements is presented in Table 2.

### 2.6. Statistics

A deprivation index (DI) was calculated to assess the within-animal percent difference ((nondeprived layer A1 + nondeprived layer A)‐(deprived layer A1 + deprived layer A)/(nondeprived layer A1 + nondeprived layer A)) in neuronal somal size and density of neurofilament immunoreactivity between deprived- and non-deprived-eye layers [43, 49, 50]. All statistical analyses and data visualizations were performed using Prism (GraphPad, USA). Statistical comparisons between deprived and nondeprived layers within each rearing condition were performed using Mann-Whitney *U* tests. Statistical comparisons between rearing conditions were made using Permutation tests for a difference in means, and we applied the Benjamini-Hochberg procedure for controlling false discovery rate (6 total comparisons). Adjusted *p* values are reported [51].

## 3. Results

### 3.1. Within Condition Effects

**Figure 2:**
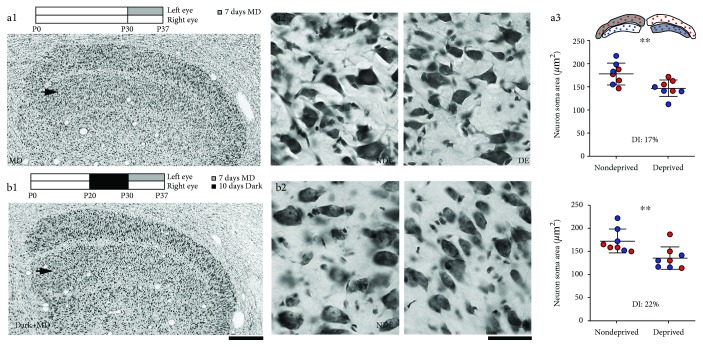
The effect on neuron size of a 7-day duration of MD imposed at the peak of the critical period with and without a prior 10-day period of darkness. Schematic at the top of (a1) and (b1) indicate the rearing history and timeline of procedures for each group. The effect of 7 days of MD imposed at postnatal day 30 was obvious upon gross examination of the eye-specific layers of the dLGN (a1), as well as at higher magnification where neurons within deprived-eye (DE) layers were smaller than neurons within non-deprived-eye (NDE) counterpart layers. Stereological quantification of soma size revealed that deprived neurons were rendered 17% smaller than nondeprived neurons, which represented a significant difference. When the same MD was imposed immediately following 10 continuous days of dark exposure started at postnatal day 20, there was an obvious reduction in the staining intensity within deprived-eye layers compared to non-deprived-eye layers (b1). The paler staining within deprived-eye layers was accompanied by a reduction in the size of deprived neurons when compared to nondeprived neurons (b2). Quantification of neuron cross-sectional soma size revealed that deprived neurons were significantly smaller than nondeprived neurons by an average of 22%. Drawing in (a3) represents eye-specific layers of the dLGN with red and blue circles indicating measurements from A and A1 layers, respectively. Scale bars = 500 *μ*m (1) and 50 *μ*m (2). Arrows in (a1) and (b1) indicate the deprived-eye layer. Asterisks indicate statistical significance at probability < 0.05.

Low-power (4x objective) microscopic examination of Nissl-stained dLGN sections from animals in the 7-day MD-only group revealed a clear deprivation effect characterized by smaller neuron somata and reduced staining intensity within deprived-eye layers (Figure 2(a), a1 and a2; Table 2). Nissl-stained sections of dLGN from animals that received 10 days of darkness before 7 days of MD also showed an obvious reduction in the size of neuron somata that was accompanied by a loss of staining intensity within deprived-eye layers (Figure 2(b), b1 and b2). From our initial low-power observations of staining intensity reduction induced by MD, we noted that the contrast between deprived and nondeprived layers appeared slightly greater in the group subjected to darkness before MD, suggestive of a larger deprivation effect relative to the MD-only group; this difference was also revealed by our *Between Condition* analysis below. Observations of the anatomical differences that were evident at low magnification were reflected in the quantification of the cross-sectional area of somata from both groups. In the MD-only group, there was a clear difference in the average size of deprived relative to nondeprived neurons, with deprived neurons (average = 147.8 *μ*m^2^; SD = 16 *μ*m^2^) 17% smaller than nondeprived neurons (average = 178.8 *μ*m^2^; SD = 23 *μ*m^2^; Figure 2(a), a3). Deprived neurons were significantly smaller than nondeprived neurons in this MD-only group (*p* < 0.02; Mann-Whitney *U* test; *n* = 16 layers). In the group of animals that received darkness prior to MD, the size of deprived neurons (average = 134.5 *μ*m^2^; SD = 21 *μ*m^2^) was also measured to be smaller, by an average of 22%, compared to nondeprived neurons (average = 172.5 *μ*m^2^; SD = 26 *μ*m^2^; Figure 2(b), b3). Statistical analysis revealed this difference was also significant (*p* < 0.02; Mann-Whitney *U* test;  *n* = 16 layers).

**Figure 3:**
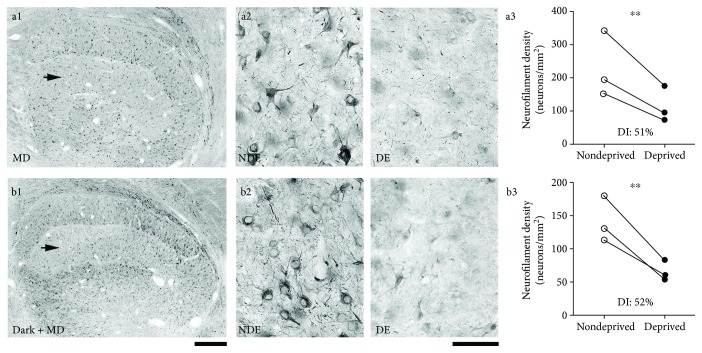
Examination of the magnitude of neurofilament loss in the dLGN from animals that were either monocularly deprived for 7 days at the peak of the critical period or else received 10 days of dark exposure before the same length of MD. Monocular deprivation alone produced a substantial reduction in the amount of neurofilament labeling within deprived-eye dLGN layers (a1). At high magnification, the loss of labeling was evident as a reduced number of immunopositive neurons, as well as a reduction in labeling intensity (a2) in deprived-eye (DE) relative to non-deprived-eye (NDE) layers. When the same MD was preceded by 10 days of darkness, a similar reduction in neurofilament within deprived-eye layers was observed at low (b1) as well as at high magnification (b2). Stereological quantification of the density of neurofilament-positive neurons revealed that MD-only (a3) and MD preceded by dark exposure (b3) produced a similar deprivation effect, each with a significant reduction in deprived layers. Arrows in (a1) and (b1) indicate the deprived-eye layer. Asterisks indicate statistical significance at probability < 0.05. Scale bars = 500 *μ*m (1) and 50 *μ*m (2).

Similar to the reduction of soma size precipitated by MD, loss of neurofilament protein in the dLGN has emerged as a sensitive means of measuring the effect of visual deprivation [52, 53]. In the current study, we observed a considerable loss of neurofilament labeling in the dLGN following MD for 7 days (Figure 3(a), a1–a3), with deprived layers having significantly fewer (*p* < 0.02; Mann-Whitney *U* test; *n* = 12 layers) immunopositive neurons (average = 115 neurons/mm^2^; SD = 54 neurons/mm^2^) compared to nondeprived layers (average = 230 neurons/mm^2^; SD = 99 neurons/mm^2^), which corresponded to a 51% reduction in immunopositive cells. Animals that received darkness prior to MD also showed a strong deprivation effect (Figure 3(b), b1–b3), with deprived layers having significantly fewer (*p* < 0.02; Mann-Whitney *U* test; *n* = 12 layers) immunopositive neurons (average = 67 neurons/mm^2^; SD = 15 neurons/mm^2^) relative to nondeprived layers (average = 142 neurons/mm^2^; SD = 35 neurons/mm^2^), which corresponded to a 52% reduction in immunopositive cells.

### 3.2. Between Condition Effects

**Figure 4:**
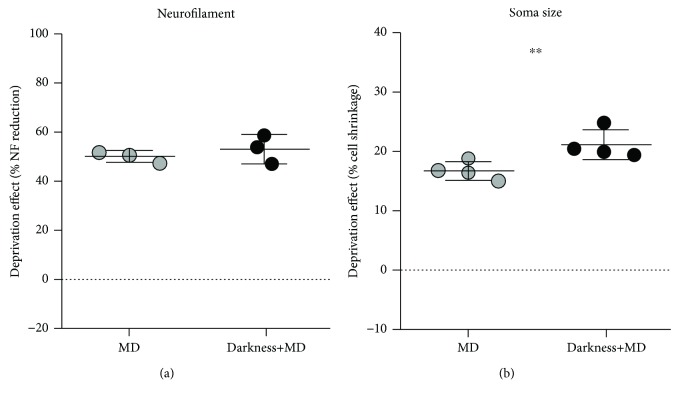
Comparison of the MD effect between MD-only and dark exposure followed by MD conditions. Whereas comparison of the effect of MD on neurofilament labeling across conditions indicated that the loss in the dLGN was similar with or without darkness (a), the effect on soma size was significantly larger in the group of animals that received dark exposure prior to MD (b). Asterisks indicate statistical significance at probability < 0.05.

Next, we sought to quantify whether the deprivation effect was greater for the group subjected to darkness before MD relative to the MD-only group. Within-animal percent differences (DI) in density of neurofilament immunoreactivity and neuron soma size were used to test for group effects between the MD-only condition and darkness prior to MD condition. DIs were similar for neurofilament immunoreactivity between the MD-only condition and darkness prior to MD condition (Figure 4(a)), indicating that on this measure dark exposure did not produce an exaggerated effect. This conclusion was supported by a statistical test of DIs between groups, which revealed that the magnitude of neurofilament loss was not significantly larger in the group that received dark exposure prior to MD (*p* = 0.4286; Permutation test; *n* = 6 cats). Conversely, when changes in soma size were compared between the two groups, the animals where darkness preceded MD showed changes that were approximately 23% larger than MD-only animals (Figure 4(b)), and this difference was significant (*p* = 0.02; Permutation test; *n* = 8 cats).

## 4. Discussion

In this study, we demonstrate that a short period of darkness preceding a week of MD initiated at the peak of the critical period can produce a modest increase in the effect of MD on the difference between deprived and nondeprived soma size in the dLGN. However, we did not observe a difference in the magnitude of neurofilament loss within deprived-eye layers between MD-only and MD following darkness conditions. The enhanced MD effect with darkness indicates that the natural peak of plasticity is malleable on some measures and suggests that brief dark exposure, unlike longer dark rearing, does not act by delaying both the onset and decay of the critical period. This result, as well as studies demonstrating enhanced plasticity following dark exposure long after the critical period has waned [39, 40, 44], is inconsistent with the suggestion that darkness slows the entire profile of the critical period but instead suggests that brief darkness can alter key plasticity parameters [54] to rejuvenate the visual system and bring about heightened plasticity capacity.

The absence of an increased effect of MD following dark exposure on neurofilament labeling may be due to neurofilament loss reaching saturation faster than the effect that MD exerts on soma size. Examination of effect sizes with shorter durations of MD may have revealed a difference between the two groups with regard to neurofilament labeling. This dichotomy between the effect of MD on neurofilament and soma size is mirrored in a study conducted on cats that examined the effect of dark exposure beyond the critical period peak, which showed a slight plasticity boost with measurements of soma size but not with changes in neurofilament labeling [45]. Shifts in cortical ocular dominance measured physiologically elicited by MD can also emerge and saturate quickly, with shifts occurring within 1-2.5 days of MD onset and saturation by about a week of MD [55–57]. Interestingly, a study that examined ocular dominance shifts in kittens that were monocularly deprived after receiving a prior period of dark rearing from birth revealed a slightly exaggerated ocular dominance shift when darkness occurred from birth to 50 days and was followed by 2.5 days of MD compared to the MD-only group (see Figure 3 in [57]); though with longer MD, there was no difference between groups.

The enhancement of plasticity observed in the group of animals subjected to dark exposure may originate from a reduction in deprived neuron size, a hypertrophy of nondeprived neuron size, or a combination of the two effects. The effect of monocular deprivation on cells within the dLGN includes both a shrinkage of neurons innervated by the deprived eye and hypertrophy of neurons connected to the nondeprived eye. The atrophy and hypertrophy of neurons in the context of monocular deprivation are both manifestations of neural plasticity. The magnitude of nondeprived neuron hypertrophy has been estimated to be about 10% [58]. The hypertrophy of nondeprived neurons likely derives from an expansion of nondeprived terminal fields, as has been demonstrated previously [5]. Darkness beginning at birth and lasting 3 weeks does not reduce the size of neurons within the dLGN; however, further dark exposure up to 12 weeks does reduce the size of dLGN neurons relative to normal controls [59]. It is possible that in our study, dark exposure for 10 days resulted in smaller dLGN neurons relative to controls, meaning that the enhanced deprivation effect may derive from nondeprived neuron hypertrophy. Examination of a darkness-only group would address this possibility. Irrespective of the size of neurons immediately following dark exposure, the enhanced DI observed when MD follows darkness demonstrates a higher level of neural plasticity compared to the MD-only condition.

In rodents, the enhancement of neural plasticity produced by dark exposure is thought to partly originate from a shift in NMDA receptor subunits toward the neonatal isoform [60, 61], as well as a rejuvenation of inhibitory synaptic transmission [21]. Examination of the effects of inhibitory neural transmission and plasticity capacity following dark exposure applied at different ages has revealed in rodents a refractory period for plasticity enhancement [21]. The enhancement of ocular dominance plasticity observed in very young or adult rodents following 10 days of dark exposure is not observed during a refractory period that occurs between postnatal days 35 and 55 [21]. It is possible that such a refractory period also exists for cats that are subjected to dark exposure beyond the critical period peak, which in cats occurs at about postnatal day 30 [9]. Given that adult cats exposed to darkness do not exhibit the same enhancement in plasticity that younger cats demonstrate [62], it is alternatively possible that the cat visual system exhibits a progressive decline in the capacity for dark exposure to enhance plasticity in the visual system.

The natural peak of the critical period for ocular dominance plasticity emerges as a consequence of a molecular balance between plasticity facilitators and inhibitors. While the stages of development and maturity of the visual system are characterized by a changing landscape of molecules [63, 64], it appears that dark exposure can have an effect across a broad collection of molecular arrangements and does not seem to effect influence upon a single molecular conglomeration. This confers broad applicability to dark exposure as a means of promoting plasticity at various stages during postnatal development. Although dark exposure imposed in adult cats does not produce elevated plasticity levels [62], dark exposure is efficacious at ages past the critical period peak [41, 45], and results from the current study demonstrate that elevated plasticity can also be elicited very early in postnatal development when plasticity is naturally at its highest.

That the natural peak of the critical period is modifiable through dark exposure raises the intriguing possibility that darkness could be used as an auxiliary to gold standard treatments for human amblyopia with the aim of expediting the recovery of visual function and perhaps producing superior outcomes for vision. The use of dark exposure in conjunction with other treatments for amblyopia such as occlusion therapy, perceptual learning, or video game play may provide a means of enhancing recovery outcomes early in development and not just when the efficacy of conventional treatments fades with age. Visual training has emerged as a robust approach to promote recovery from amblyopia in rats, cats, and humans [65–68], and a recent rat study has examined recovery outcomes when dark exposure was immediately followed by visual training in rodents [68]. Visual training that quickly followed dark exposure promoted recovery from severe amblyopia in rats, whereas amblyopia was not reversed with visual training alone [68]. It was suggested that this form of recovery occurs in a two-step process that involves a reactivation of synaptic plasticity mediated by dark exposure, followed by visual training that instructs synaptic modifications and promotes visual recovery. The plasticity enhancement that we demonstrate in the current study raises the possibility that such combinatorial therapy may prove beneficial not only when applied beyond the critical period but also when applied at younger ages that may benefit from an increase in the speed and or amount of recovery.

## Figures and Tables

**Table 1:** Antibody characterization.

Antigen	Immunogen	Source	Dilution
Neurofilament H	Homogenized rat hypothalamus	Covance (Princeton, NJ), mouse monoclonal, clone SMI-32, No. SMI-32. AB_509998	1 : 1000

**Table 2:** Measurements of the average soma area (*μ*m^2^) and neurofilament-positive cell density (cells/mm^2^) presented for animals across both rearing conditions in this study. Deprivation index (DI) represents the percentage difference between deprived and nondeprived layers for each animal studied. Asterisks indicate measurements that were not collected because immunolabelling was insufficient for quantification.

Cat #	Condition	Soma area nondeprived	Soma area deprived	DI (%)	Neurofilament nondeprived	Neurofilament deprived	DI (%)
#100	MD	180	150	16	342	176	49
#101	MD	176	145	17	195	95	51
#102	MD	208	168	19	153	73	52
#103	MD	151	128	15	^∗^	^∗^	^∗^
#110	DR+MD	162	129	21	114	61	46
#111	DR+MD	211	166	22	181	84	54
#112	DR+MD	165	123	25	131	56	57
#113	DR+MD	152	120	21	^∗^	^∗^	^∗^

## Data Availability

The data used to support the findings of this study are available from the corresponding author upon request.
